# Individuals Treated for Gender Dysphoria with Medical and/or Surgical Transition Who Subsequently Detransitioned: A Survey of 100 Detransitioners

**DOI:** 10.1007/s10508-021-02163-w

**Published:** 2021-10-19

**Authors:** Lisa Littman

**Affiliations:** The Institute for Comprehensive Gender Dysphoria Research, 489 Main Street, Warren, RI 02885 USA

**Keywords:** Gender dysphoria, Detransition, Transgender

## Abstract

The study’s purpose was to describe a population of individuals who experienced gender dysphoria, chose to undergo medical and/or surgical transition and then detransitioned by discontinuing medications, having surgery to reverse the effects of transition, or both. Recruitment information with a link to an anonymous survey was shared on social media, professional listservs, and via snowball sampling. Sixty-nine percent of the 100 participants were natal female and 31.0% were natal male. Reasons for detransitioning were varied and included: experiencing discrimination (23.0%); becoming more comfortable identifying as their natal sex (60.0%); having concerns about potential medical complications from transitioning (49.0%); and coming to the view that their gender dysphoria was caused by something specific such as trauma, abuse, or a mental health condition (38.0%). Homophobia or difficulty accepting themselves as lesbian, gay, or bisexual was expressed by 23.0% as a reason for transition and subsequent detransition. The majority (55.0%) felt that they did not receive an adequate evaluation from a doctor or mental health professional before starting transition and only 24.0% of respondents informed their clinicians that they had detransitioned. There are many different reasons and experiences leading to detransition. More research is needed to understand this population, determine the prevalence of detransition as an outcome of transition, meet the medical and psychological needs of this population, and better inform the process of evaluation and counseling prior to transition.

**Supplementary Information:**

The online version contains supplementary material available at 10.1007/s10508-021-02163-w.

## Introduction

Detransition is the act of stopping or reversing a gender transition. The visibility of individuals who have detransitioned is new and may be rapidly growing. As recently as 2014, it was challenging for an individual who detransitioned to find another person who similarly detransitioned (Callahan, 2018). Between 2015 and 2017, a handful of blogs written by individual detransitioners started to appear online, private support groups for detransitioners formed, and interviews with detransitioners began to appear in news articles, magazines, and blogs (Anonymous, 2017; 4thwavenow, 2016; Herzog, 2017; McCann, 2017). Although few YouTube videos about detransition existed prior to 2016, multiple detransitioners started to post videos documenting their experiences in 2016 and the numbers of these videos continues to increase.^1^ In late 2017, the subreddit r/detrans (r/detrans, 2020) was revitalized and in four years has grown from 100 members to more than 21,000 members. A member poll of r/detrans conducted in 2019 estimated that approximately one-third of the members responding to the survey were desisters or detransitioners (r/detrans, 2019). The Pique Resilience Project, a group of four detransitioned or desisted young women, was founded in 2018 as a way to share the experiences of detransitioners with the public (Pique Resilience Project, 2019). In late 2019, the Detransition Advocacy Network, a nonprofit organization to “improve the well-being of detransitioned people everywhere” was launched (The Detransition Advocacy Network, 2020) and the first formal, in-person conference for detransitioned people was held (Bridge, 2020). In the face of this massive change, clinicians have called for more research into the experiences of detransitioners (Butler & Hutchinson, 2020; Entwistle, 2021; Marchiano, 2020).

Although there were rare published reports about detransitioners prior to 2016, most of the published literature about detransition is recent (Callahan, 2018; D’Angelo, 2018; Djordjevic et al., 2016; Kuiper & Cohen-Kettenis, 1998; Levine, 2018; Marchiano, 2017; Pazos Guerra et al., 2020; Stella, 2016; Turban & Keuroghlian, 2018; Turban et al., 2021; Vandenbussche, 2021). The prevailing cultural narratives about detransition are that most individuals who detransition will retransition and that the reasons for detransition are discrimination, pressures from others, and nonbinary identification (Turban et al., 2021). However, case reports are shedding light on a broader and more complex range of experiences that include trauma, worsened mental health with transition, re-identification with natal sex, and difficulty separating sexual orientation from gender identity (D’Angelo, 2018; Levine, 2018; Pazos Guerra et al., 2020).^2^ Detransitioners and desisters, in their own words, have provided additional depth to the discussion, describing that:

1. Trauma (including sexual trauma) and mental health conditions contributed to their transgender identification and transition (Callahan, 2018; Herzog, 2017; twitter.com/ftmdetransed & twitter.com/radfemjourney, 2019)
2. Their dysphoria and transition were due to homophobia and difficulty accepting themselves as homosexual (Bridge, 2020; Callahan, 2018; upperhandMARS, 2020)
3. Peers, social media, and online communities were influential in the development of transgender identification and desire to transition (Pique Resilience Project, 2019; Tracey, 2020; upperhandMARS, 2020)
4. Their dysphoria was rooted in misogyny (Herzog, 2017)

Two recently published convenience sample reports provide additional context about the topic of detransition. First, Turban et al. (2021) analyzed data from the United States Trans Survey (USTS) (James et al., 2016). The USTS contains data from 27,715 transgender and gender diverse adults from the U.S. who were recruited through lesbian, gay, bisexual, transgender, queer (LGBTQ), and allied organization outreach. The USTS included the question, “Have you ever detransitioned? In other words, have you ever gone back to living as your sex assigned at birth, at least for a while?” with the multiple choice options of “yes,” “no,” and “I have never transitioned.” For the 2,242 participants who answered “yes,” Turban et al. analyzed the responses to the multiple choice question, “Why did you de-transition? In other words, why did you go back to living as your sex assigned at birth? (Mark all that apply).” Although most of the offered answer options were about external pressures to detransition (pressure from spouse or partner, pressure from family, pressure from friends, pressure from employer, discrimination, etc.), participants could write in additional reasons that were not listed. Turban et al.’s sample included more natal males (55.1%) than natal females (44.9%). Roughly half (50.2%) had taken cross-sex hormones and 16.5% had obtained surgery. The findings revealed that most (82.5%) of the sample expressed at least one external factor for detransitioning and 15.9% expressed at least one internal factor (factors originating from self).

The second study by Vandenbussche (2021) recruited detransitioners from online communities of detransitioners and analyzed data for the participants who answered affirmatively to the question, “Did you transition medically and/or socially and then stopped?” The sample of 237 participants was predominantly natal female (92%), and from the U.S. (51%) and Europe (32%). Most (65%) had transitioned both medically and socially. Participants selected from multiple choice options to indicate why they detransitioned with options covering a range of experiences. Respondents also had the option to write in additional reasons. Frequently endorsed reasons for detransition included realizing that their gender dysphoria was related to other issues (70%); health concerns (62%); observing that transition did not help their dysphoria (50%); and that they found alternatives to deal with their dysphoria (45%). In contrast to Turban et al. (2021), external factors such as lack of support, financial concerns, and discrimination were less common (13%, 12%, and 10%, respectively). Many in the sample described that when they detransitioned they lost support or were ostracized from lesbian, gay, bisexual, and transgender (LGBT) communities, suggesting that many of the participants in Vandenbussche (2021) would not have been reached by the recruitment efforts of the USTS (James et al., 2016).

The objective of the current study was to describe a population of individuals who experienced gender dysphoria, chose to undergo medical and/or surgical transition and then detransitioned by discontinuing medications, having surgery to reverse the effects of transition, or both. In contrast to Turban et al. (2021) and Vandenbussche (2021), this study focused only on individuals who transitioned and detransitioned medically, surgically, or both. For the purpose of this study, medical transition refers to the use of puberty blockers, cross-sex hormones, or anti-androgens and surgical transition refers to any of a variety of surgical procedures (common surgical procedures include mastectomy, genital surgery, and breast augmentation). This study does not describe the population of individuals who undergo medical or surgical transition without issue nor is it designed to assess the prevalence of detransition as an outcome of transition. Instead, the goal was to identify detransition reasons and narratives in order to inform clinical care and future research.

## Method

### Participants and Procedure

During the recruitment period, 101 individuals who met the study criteria completed online surveys. Inclusion criteria were (1) completion of a survey via Survey Monkey; (2) answering that they had taken or had one or more of the following for the purpose of gender transition: cross-sex hormones, anti-androgens, puberty blockers, breast surgery, genital surgery, other surgery; and (3) answering that they had done any of the following for the purpose of detransitioning: stopped taking cross-sex hormones, stopped taking anti-androgens, stopped taking puberty blockers, had any surgery to reverse transition. One survey was excluded for nonsense answers leaving 100 surveys for analysis. The sample included more natal females (69.0%) than natal males (31.0%) with respondents who were predominantly White (90.0%), non-Hispanic (98.0%), resided in the U.S. (66.0%); had no religious affiliation (63.0%), and support the rights of gay and lesbian couples to marry legally (92.9%) (see Table 1). At the time of survey completion, the mean age of respondents was 29.2 years (SD = 9.1) though natal females were significantly younger (*M* = 25.8; SD = 5.0) than natal males (*M* = 36.7; SD = 11.4), *t*(98) = − 6.56, *p* < .001. Prior to transitioning, natal females were more likely to report an exclusively homosexual sexual orientation and natal males were more likely to report an exclusively heterosexual sexual orientation.

**Table 1:** Demographic and baseline characteristics

	Natal female *N* (%) *N* = 69	Natal male *N* (%) *N* = 31
Race/ethnicity*		
White	62 (89.9%)	28 (90.3%)
Multiracial	6 (8.7%)	3 (9.7%)
Other	4 (5.8%)	0 (0%)
Asian	1 (1.4%)	1 (3.2%)
Hispanic	1 (1.4%)	1 (3.2%)
Black	0 (0%)	0 (0%)
*Country of residence*		
USA	46 (66.7%)	20 (64.5%)
UK	8 (11.6%)	1 (3.2%)
Canada	5 (7.2%)	4 (12.9%)
Australia	2 (2.9%)	2 (6.5%)
Other	8 (11.6%)	4 (12.9%)
*Education*		
Bachelor’s or graduate degree	29 (42.0%)	18 (58.1%)
Associates degree	3 (4.3%)	1 (3.2%)
Some college but no degree	28 (40.6%)	9 (29.0%)
High school graduate or GED	8 (11.6%)	2 (6.5%)
< High school	1 (1.4%)	0 (0%)
Other	0 (0%)	1 (3.2%)
*Socioeconomic status compared to others in country of residence*		
Above average (somewhat or very much)	19 (27.5%)	12 (38.7%)
About average	20 (29.0%)	7 (22.6%)
Below average (somewhat or very much)	27 (39.1%)	12 (38.7%)
Prefer not to say	3 (4.3%)	0 (0%)
*Categorized sexual orientation (by natal sex) prior to transition*^a^		
Homosexual	18 (26.1%)	2 (6.5%)
Heterosexual	6 (8.7%)	12 (38.7%)
Bisexual	15 (21.7%)	8 (25.8%)
Pansexual	4 (5.8%)	1 (3.2%)
Multiple	20 (29.0%)	5 (16.1%)
Asexual	6 (8.7%)	3 (9.7%)
*Religious affiliation*		
No religious affiliation	41 (59.4%)	22 (73.3%)
Liberal Christian	5 (7.2%)	3 (10.0%)
Liberal Jewish	5 (7.2%)	0 (0%)
Conservative Christian	1 (1.4%)	2 (6.7%)
Liberal Muslim	1 (1.4%)	0 (0%)
Conservative Jewish	0 (0%)	0 (0%)
Conservative Muslim	0 (0%)	0 (0%)
Other	16 (23.2%)	3 (10.0%)
*Legal marriage for gay and lesbian couples*		
Favor	65 (97.0%)	26 (83.9%)
Oppose	1 (1.5%)	5 (16.1%)
Don’t know	1 (1.5%)	0 (0%)
*Source where participant first heard about study*		
Detransition blogs	26 (37.7%)	15 (48.4%)
Other social media	37 (53.6%)	11 (35.5%)
A person they know	3 (4.3%)	3 (9.7%)
Other	3 (4.3%)	2 (6.5%)

*May select more than one answer

^a^Natal females were more likely to express an exclusively homosexual sexual orientation prior to transition (*χ*^2^ = 5.15. The *p*-value is .023). Natal males were more likely to express an exclusively heterosexual sexual orientation prior to transition (*χ*^2^ = 13.05. The *p* value is < .001). Natal sex differences were not significant for individuals expressing pre-transition sexual orientations of bisexual, pansexual, multiple, and asexual. For bisexual sexual orientation, *χ*^2^ = 0.20. For pansexual sexual orientation, *χ*^2^ = 0.29. For multiple sexual orientations reported, *χ*^2^ = 1.88. For asexual sexual orientation, *χ*^2^ = 0.02

A 115-question survey instrument with multiple choice, Likert-type, and open-ended questions was created by the author and two individuals who had personally detransitioned. The author had met both detransitioners by way of introductions from colleagues. The author and both individuals who had detransitioned created questions for the survey, provided feedback, and revised the survey questions collaboratively with a focus on content, clarity, and relevance to a variety of transition and detransition experiences. The survey instrument included two questions that were adapted from an online survey of female detransitioners (Stella, 2016). Once completed, the survey was uploaded onto Survey Monkey (SurveyMonkey, Palo Alto, CA) via an account that was HIPAA-enabled.

Recruitment information with a link to the survey was posted on blogs that covered detransition topics and shared in a private online detransition forum, in a closed detransition Facebook group, and on Tumblr, Twitter, and Reddit. Recruitment information was also shared on the professional listservs for the World Professional Association for Transgender Health, the American Psychological Association Section 44, and the SEXNET listserv (which is a listserv of sex researchers and clinicians) and the professionals on the listservs were asked to share recruitment information with anyone they knew who might be eligible. Efforts were made to reach out to communities with varied views about the use of medical and surgical transition and recruitment information stated that participation was sought from individuals regardless of whether their transition experiences were positive, negative or neutral. Potential participants were invited to share recruitment information with any potentially eligible person or community with potentially eligible people. The survey was active from December 15, 2016 to April 30, 2017 (4.5 months). The median time to complete a survey was 49 min; 50% of the surveys were completed between 32 and 71 min. There were no incentives offered for participating. Data were collected anonymously, without IP addresses, and stored securely with Survey Monkey.

Participation in this study was voluntary. Electronic consent was obtained from all participants in the following manner. The first page of the online survey informed respondents about the research purpose, potential risks and benefits, that participation was voluntary, and provided contact information for the researcher. Survey questions were only displayed if the participant clicked “agree” which indicated that they read the information, voluntarily agreed to participate and were at least 18 years of age.

### Measures

#### Demographic and Baseline Characteristics

Information was collected about participant age, natal sex, race/ethnicity, country of residence, educational attainment, socioeconomic status, religion, attitudes about legal marriage for gay and lesbian couples, and where they first heard about the study. The term sexual orientation in this article is intended to refer to the natal sex of the participant and the natal sex of the individuals with whom they are sexually attracted. Participants were asked to select one or more labels for how they identified their sexual orientation prior to transition with options inclusive of participant sex (e.g., asexual female, bisexual female, heterosexual female, etc.). These responses were coded to be consistent with participant natal sex and were categorized into homosexual, heterosexual, bisexual, pansexual, asexual, and multiple. The multiple category included respondents who selected more than one response where responses indicated more than one pattern of sexual attraction (e.g., lesbian female and heterosexual female). Other questions about baseline characteristics included questions about diagnosed psychiatric disorders and neurodevelopmental disabilities, trauma, and non-suicidal self-injury (NSSI) before the onset of gender dysphoria.

#### Gender Dysphoria Onset and Typologies

Participants were asked how old they were when they first experienced gender dysphoria and whether this was during childhood, at the onset of puberty, during puberty, or later. Respondents were categorized as having early-onset gender dysphoria if they indicated that their gender dysphoria began “during childhood” and late-onset gender dysphoria if their gender dysphoria began “at the onset of puberty” or later. To evaluate typologies, participants were characterized by Blanchard’s (1985, 1989) typology as homosexual (if the sexual orientations listed prior to transition were exclusively homosexual) or non-homosexual which includes heterosexual, asexual, bisexual, pansexual, and multiple responses.

#### Transition

Participants were asked for their age and the year that they first sought care to transition, sources that encouraged them to believe that transition would be helpful to them, and whether they felt pressured to transition. The friendship group dynamics that were identified in previous work were assessed by asking respondents whether their friendship group mocked people who were not transgender, whether people in their pre-existing friend group transitioned before the participant decided to transition, and how participant popularity changed after announcing that they would transition (Littman, 2018). Questions were asked about participant experiences with clinicians, the social, medical, and surgical steps they took to transition, and the duration of time spent taking each medication.

#### Detransition

Participants were asked for their age and the year that they decided to detransition, how long they were transitioned before deciding to detransition, their reasons for wanting to detransition, what sources encouraged them to believe that detransition would be helpful to them, and whether they felt pressured to detransition. Participants were also asked which social, medical, and surgical steps they took to detransition and whether they contacted the doctor or clinic that they used for their transition to tell them that they detransitioned.

#### Transition and Detransition Narratives

In this article, “narratives” denote participant interpretations of their experiences and rationales surrounding their decisions to transition and detransition. To associate each participant survey with a set of relevant narratives, the data were reviewed with horizontal (beginning to end) passes and vertical passes for selected questions (these questions are listed in the supplemental materials). Surveys were coded as belonging to zero or more of the following narrative categories: discrimination, nonbinary, retransition, trauma and mental health, internalized homophobia, social influence, and misogyny. Each narrative and the responses that were associated with them are detailed below. Example quotes were selected with care taken to avoid quoting a participant more than once per narrative. Narratives are ordered and reported with the more commonly accepted narratives first and the newer narratives next.

The *discrimination* narrative was defined as when someone detransitioned due to experiencing discrimination or external social pressures. The *nonbinary* narrative consisted of answering that their current identification was “nonbinary/genderqueer” or providing open-text responses that described aspects of discovering or maintaining a nonbinary identification. Although there were no questions in the survey specifically asking about retransition, the *retransition* narrative was identified if participants expressed that they had retransitioned or resumed transition in any of the open-text responses in the survey. The *gender dysphoria was caused by trauma or a mental health condition* narrative was identified by selection for the answers, “what I thought were feelings of being transgender were actually the result of trauma,” “what I thought were feelings of being transgender were actually the result of a mental health condition,” “I discovered that my gender dysphoria was caused by something specific (ex. trauma, abuse, mental health condition)” or open-text responses consistent with these reasons. The *internalized homophobia/difficulty accepting oneself as a lesbian female, gay male, or bisexual person* narrative consisted of descriptions that the respondents’ discomfort and distress about being lesbian, gay, or bisexual was related to their gender dysphoria, transition, or detransition, or that they assumed they were transgender because they did not yet understand themselves to be lesbian, gay or bisexual. The *social pressure to transition* narrative was identified with an affirmative answer to whether they felt pressured to transition with an open-text response indicating that the pressure came from a person or group of people. The *misogyny* narrative was identified for natal female respondents with open-text responses using the word “misogyny” or expressing a hatred of femaleness.

#### Gender Identification at Start of Transition and at Survey Completion

Participants were asked how they identified their gender when they started their transition and at the time of survey completion. They were given options of female, male, nonbinary/genderqueer, trans man/FTM, trans woman/MTF, none of the above, and other. Responses were coded by natal sex and categorized as transgender, birth sex, nonbinary, and other. Answers that were combinations of the above categories were reported as combinations such as “birth sex and nonbinary.”

#### Self-Appraisal of Transition and Detransition

One question asked if participants believe they were helped and another if they were harmed by their transition with options of “very much,” “a little,” or “not at all.” These results were categorized into exclusively helped, exclusively harmed, and both helped and harmed. Participants were asked which of the following reflected their feelings about their transition: “I am glad that I transitioned,” “I wish I had never transitioned,” “Transitioning distracted me from what I should have been doing,” “Transition was a necessary part of my journey.” Participants were asked to rate their regret about their transition (“no regrets,” “mild regrets,” “strong regrets,” and “very strong regrets”) and were asked to indicate their satisfaction with their decisions to transition and detransition (“extremely satisfied,” “very satisfied,” “somewhat satisfied,” “somewhat dissatisfied,” “very dissatisfied,” and “extremely dissatisfied”). Satisfaction options were collapsed into “satisfied” and “dissatisfied.” In addition, participants were asked if they knew then what they know now, would they have chosen to transition.

### Data Analysis

After data were cleaned, statistical analyses were performed using google sheets. Results are presented as frequencies, percentages, medians, means and standard deviations. *t *tests and chi-square tests were performed for selected variables and were considered significant for *p* < .05. Qualitative data were obtained from the open-text answers to questions that allowed participants to provide additional information. Selected open-text responses were categorized, tallied, and reported numerically. Salient respondent quotes and summaries from the qualitative data were selected to illustrate the quantitative results and to provide relevant examples.

## Results

### Before Transition

Mental health diagnoses and traumatic experiences before the onset of gender dysphoria. Table 2 shows data about psychiatric disorders, neurodevelopmental disabilities, NSSI, and trauma that were reported as occurring prior to the onset of gender dysphoria. Because these conditions and events occurred before participants began to feel gender dysphoric, they cannot be considered to be secondary to gender incongruence or transphobia.

**Table 2:** Mental health diagnoses and traumatic experiences prior to the onset of gender dysphoria

	Natal female *N* (%) *N* = 69	Natal male *N* (%) *N* = 31
Diagnosed with a mental illness or neurodevelopmental disability*^a^		
Depression	27 (39.1%)	5 (16.1%)
Anxiety	22 (31.9%)	5 (16.1%)
Attention deficit hyperactivity disorder (ADHD)	10 (14.5%)	2 (6.5%)
Post-traumatic stress disorder (PTSD)	10 (14.5%)	1 (3.2%)
Eating disorders	10 (14.5%)	0 (0%)
Autism spectrum disorders	9 (13.0%)	1 (3.2%)
Bipolar disorder	9 (13.0%)	0 (0%)
Obsessive compulsive disorder	6 (8.7%)	3 (9.7%)
Borderline personality disorder	5 (7.2%)	0 (0%)
Schizophrenia or other psychotic disorders	1 (1.4%)	0 (0%)
None of the above	28 (40.6%)	17 (54.8%)
Other	7 (10.1%)	2 (6.5%)
*Non-suicidal self-injury (NSSI)*^b^		
Engaged in NSSI before the onset of gender dysphoria	19 (27.5%)	5 (16.1%)
*Trauma*^c^		
Experienced a trauma less than one year before the start of gender dysphoria	33 (47.8%)	4 (12.9%)

*May select more than one answer

^a^Natal sex difference for one or more pre-existing diagnoses (100-none of the above) was not significant [*χ*^2^(1, 100) = 1.76]

^b^Natal sex differences for NSSI before the onset of gender dysphoria was not significant (*χ*^2^ = 1.52)

^c^Experiencing a trauma less than one year before the start of gender dysphoria was statistically different [*χ*^2^(1, 100) = 11.19, *p* < .001] with natal females > natal males

Gender dysphoria onset and typology. Most participants (82.0%) were living with one or both parents when they first experienced gender dysphoria at a mean age of 11.2 years (SD = 5.6). The mean age of gender dysphoria onset was not statistically different between natal females (*M* = 11.3; SD = 5.4) and natal males (*M* = 11.0; SD = 5.9), *t*(96) = 0.25. By Blanchard typologies, 26.1% of natal females were exclusively homosexual and 73.9% non-homosexual while 6.5% of natal males were exclusively homosexual and 93.5% non-homosexual (Blanchard, 1985, 1989). Slightly more than half of the respondents (56.0%) experienced early-onset gender dysphoria and slightly less than half (44.0%) experienced late-onset gender dysphoria. Although late-onset gender dysphoria in natal females was largely absent from the scientific literature prior to 2012 (Steensma et al., 2013; Zucker & Bradley, 1995; Zucker et al., 2012a), 55.1% of the natal female participants reported that their gender dysphoria began with puberty or later. Because the information about the timing of gender dysphoria onset was obtained from participants reporting on their own experiences, it can be assumed that these cases were indeed late-onset rather than early-onset gender dysphoria that was concealed from parents and other people.

Transition reasons. Table 3 shows data about the reasons that individuals wanted to transition and the most frequently endorsed were: wanting to be perceived as the target gender (77.0%); believing that transitioning was their only option to feel better (71.0%); the sensation that their body felt wrong the way it was (71.0%), and not wanting to be associated with their natal sex (70.0%). Most participants believed that transitioning would eliminate (65.0%) or decrease (63.0%) their gender dysphoria and that with transitioning they would become their true selves (64.0%).

**Table 3:** Transition reasons

	Natal female *N* (%) *N* = 69	Natal male *N* (%) *N* = 31
Reasons for transition*		
I wanted others to perceive me as the target gender	53 (76.8%)	24 (77.4%)
I thought transitioning was my only option to feel better	50 (72.5%)	21 (67.7%)
My body felt wrong to me the way it was	50 (72.5%)	21 (67.7%)
I didn’t want to be associated with my natal sex/natal gender	51 (73.9%)	19 (61.3%)
It made me uncomfortable to be perceived romantically/sexually as a member of my natal sex/natal gender	49 (71.0%)	18 (58.1%)
I thought transitioning would eliminate my gender dysphoria	43 (62.3%)	22 (71.0%)
I felt I would become my true self	42 (60.9%)	22 (71.0%)
I identified with the target gender	40 (58.0%)	24 (77.4%)
I thought transitioning would lessen my gender dysphoria	45 (65.2%)	18 (58.1%)
I felt I would fit in better with the target gender	36 (56.5%)	20 (64.5%)
I felt I would be more socially acceptable as a member of the target gender	38 (55.1%)	11 (35.5%)
I felt I would be treated better if I was perceived as the target gender	35 (50.7%)	14 (45.2%)
I saw myself as a member of the target gender	31 (44.9%)	18 (58.1%)
I thought transitioning would reduce gender-related harassment or trauma I was experiencing	35 (50.7%)	5 (16.1%)
I had erotic reasons for wanting to transition	9 (13.0)	12 (38.7%)
Other	9 (13.0%)	3 (9.7%)

*May select more than one answer

Sources of transition encouragement and friend group dynamics. Participants identified sources that encouraged them to believe transitioning would help them. Social media and online communities were the most frequently reported, including YouTube transition videos (48.0%), blogs (46.0%), Tumblr (45.0%), and online communities (43.0%) (see supplemental materials). Also common were people who the respondents knew offline such as therapists (37.0%); someone (28.0%) or a group of friends (27.0%) that they knew in-person. A subset of participants experienced the friendship group dynamics identified in previous work, including belonging to a friendship group that mocked people who were not transgender (22.2%), having one or more friend from the pre-existing friend group transition before the participant decided to transition (36.4%), and experiencing an increase in popularity after announcing plans to transition (19.6%) (Littman, 2018). Most did not have this experience (68.7%, 61.6%, and 62.9%, respectively).

Pressure to transition. More than a third of the participants (37.4%) felt pressured to transition. Natal sex differences in feeling pressured to transition were significant by chi-square test with natal females > natal males *χ*^2^(1, 99) = 4.22, *p* = .04. Twenty-eight participants provided open-text responses of which 24 described sources of pressure (17 described social pressures and 7 described sources that were not associated with other people). Clinicians, partners, friends, and society were named as sources that applied pressure to transition, as seen in the following quotes: “My gender therapist acted like it [transition] was a panacea for everything;” “[My] [d]octor pushed drugs and surgery at every visit;” “I was dating a trans woman and she framed our relationship in a way that was contingent on my being trans;” “A couple of later trans friends kept insisting that I needed to stop delaying things;” “[My] best friend told me repeatedly that it [transition] was best for me;” “The forums and communities and internet friends;” “By the whole of society telling me I was wrong as a lesbian;” and “Everyone says that if you feel like a different gender…then you just are that gender and you should transition.” Participants also felt pressure to transition that did not involve other people as illustrated by the following: “I felt pressured by my inability to function with dysphoria” and “Not by people. By my life circumstances.”

Experiences with clinicians. When participants first sought care for their gender dysphoria or desire to transition, more than half of the participants (53.0%) saw a psychiatrist or psychologist; about a third saw a primary care doctor (34.0%) or a counselor (including licensed clinician social worker, licensed professional counselor, or marriage and family therapist) (32.0%); and 17.0% saw an endocrinologist. For transition, 45.0% of participants went to a gender clinic (44.4% of those attending a gender clinic specified that the gender clinic used the informed consent model of care); 28.0% went to a private doctor’s office; 26.0% went to a group practice; and 13.0% went to a mental health clinic (see supplemental materials).

The majority (56.7%) of participants felt that the evaluation they received by a doctor or mental health professional prior to transition was not adequate and 65.3% reported that their clinicians did not evaluate whether their desire to transition was secondary to trauma or a mental health condition. Although 27.0% believed that the counseling and information they received prior to transition was accurate about benefits and risks, nearly half reported that the counseling was overly positive about the benefits of transition (46.0%) and not negative enough about the risks (26.0%). In contrast, only a small minority found the counseling not positive enough about benefits (5.0%) or too negative about risks (6.0%) suggesting a bias toward encouraging transition.

### Transition

Participants were on average 21.9 years old (SD = 6.1) when they sought medical care to transition with natal females seeking care at younger ages (*M* = 20.0; SD = 4.2) than natal males (*M* = 26.0; SD = 7.5), *t*(97) = − 5.07, *p* < .001. Given that the majority of natal males were categorized as Blanchard typology non-homosexual, the finding that natal males sought medical care to transition at older ages than natal females is concordant with previous research (Blanchard et al., 1987). The average year for seeking care was more recent for natal females (*M* = 2011; SD = 3.8) than natal males (*M* = 2007; SD = 6.9), *t*(96) = 2.78, *p* = .007, and thus, there may have been differences in the care they received due to differences in the culture surrounding transition and the prevailing medical approaches to gender dysphoria for the time.

At the start of transitioning, nearly all (98.0%) of the participants identified as either transgender (80.0%), nonbinary (15.0%), or both transgender and nonbinary (3.0%). Participants identified which social, medical, and surgical steps they had taken to transition. Table 4 shows these steps, separated by natal sex where appropriate. Most respondents adopted new pronouns (91.0%) and names (88.0%), and the vast majority (97.1%) of natal females wore a binder. Most participants took cross-sex hormones (96.0%) and most natal males took anti-androgens (87.1%). The most frequent transition surgery was breast or chest surgery for natal females (33.3%). Genital surgery was less common (1.4% of natal females and 16.1% of natal males). Natal females took testosterone for a mean duration of 2.0 years (SD = 1.6). Natal males took estrogen for a mean duration of 5.1 years (SD = 5.9) and anti-androgens for 2.8 years (SD = 2.6). The minority of patients who took puberty blockers took them for a mean duration of less than a year (*M* = 0.9 years; SD = 0.6).

**Table 4:** Steps taken for social, medical, and surgical transition

	*N* (%)
Social transition*	
Pronouns	91 (91.0%)
Different name	88 (88.0%)
Clothes/hair/makeup	90 (90.0%)
Legal name change	49 (49.0%)
Gender/sex changed on government documents	36 (36.0%)
Voice training	20 (20.0%)
Natal female	
Wore a binder	67 (97.1%)
Medical transition*	
Cross-sex hormones	96 (96.0%)
Puberty blockers	7 (7.0%)
Natal male	
Anti-androgens	27 (87.1%)
Surgical transition*	
Face/neck surgery	5 (5.0%)
Natal female	
Breast/chest surgery	23 (33.3%)
Genital surgery (to create a penis)	1 (1.4%)
Natal male	
Breast implants	5 (16.1%)
Genital surgery (to create a vagina)	5 (16.1%)

*May select more than one answer

### Detransition

Before deciding to detransition, participants remained transitioned for a mean duration of 3.9 years (SD = 4.1) with natal females remaining transitioned for a shorter period of time (*M* = 3.2 years; SD = 2.7) than natal males (*M* = 5.4 years; SD = 6.1), *t*(96) = − 2.40, *p* = .018. When participants decided to detransition they were a mean age of 26.4 years old (SD = 7.4) though natal females were significantly younger (*M* = 23.6; SD = 4.5) than natal males (*M* = 32.7; SD = 8.8), *t*(97) = − 6.75, *p* < .001. The mean calendar year when participants decided to detransition was 2014 (*M* = 2014; SD = 3.3), but the difference between natal females and natal males was not significant (*M* = 2014, SD = 3.3; *M* = 2014, SD = 3.5), *t*(95) = 0.52.

Respondents detransitioned for a variety of reasons and most (87.0%) selected more than one reason. The most frequently endorsed reason for detransitioning was that the respondent’s personal definition of male and female changed and they became comfortable identifying with their natal sex (60.0%) (see Table 5). Other commonly endorsed reasons were concerns about potential medical complications (49.0%); transition did not improve their mental health (42.0%); dissatisfaction with the physical results of transition (40.0%); and discovering that something specific like trauma or a mental health condition caused their gender dysphoria (38.0%). External pressures to detransition such as experiencing discrimination (23.0%) or worrying about paying for treatments (17.0%) were less common.

**Table 5:** Reasons for detransitioning

	Natal female *N* (%) *N* = 69	Natal male *N* (%) *N* = 31
Reasons for detransitioning*		
My personal definition of female or male changed and I became more comfortable identifying as my natal sex	45 (65.2%)	15 (48.4%)
I was concerned about potential medical complications from transitioning	40 (58.0%)	9 (29.0%)
My mental health did not improve while transitioning	31 (44.9%)	11 (35.5%)
I was dissatisfied by the physical results of the transition/felt the change was too much	35 (50.7%)	5 (16.1%)
I discovered that my gender dysphoria was caused by something specific (ex, trauma, abuse, mental health condition)	28 (40.6%)	10 (32.3%)
My mental health was worse while transitioning	27 (39.1%)	9 (29.0%)
I was dissatisfied by the physical results of the transition/felt the change was not enough	22 (31.9%)	11 (35.5%)
I found more effective ways to help my gender dysphoria	25 (36.2%)	7 (22.6%)
My physical health was worse while transitioning	21 (30.4%)	11 (35.5%)
I felt discriminated against	12 (17.4%)	11 (35.5%)
I had medical complications from transitioning	12 (17.4%)	7 (22.6%)
Financial concerns about paying for transition care	11 (15.9%)	6 (19.4%)
My gender dysphoria resolved	10 (14.5%)	5 (16.1%)
My physical health did not improve while transitioning	9 (13.0%)	2 (6.5%)
I resolved the specific issue that was the cause of my gender dysphoria	6 (8.7%)	4 (12.9%)
I realized that my desire to transition was erotically motivated	1 (1.4%)	5(16.1%)
Other	19 (27.5%)	6 (19.4%)

*May select more than one answer

Encouragement and pressure to detransition. Participants were asked to select sources that encouraged them to believe that detransitioning would help them. These included blogs (37.0%), Tumblr (35.0%), and YouTube detransition videos (23.0%) (see supplemental materials). At some point in their process, 23.2% felt pressured to detransition. There was no significant difference between natal females and natal males for feeling pressured to detransition, *χ*^2^(1, 99) = 1.11. Of the 21 open-text responses provided, 14 respondents expressed social pressure to detransition; three expressed internal pressure to detransition and four provided responses that were neither or unclear. Regarding social pressure to detransition, seven participants expressed that the pressure came from partners, parents, or other family members as shown in the following example quotes: “I was threatened that if I did not immediately detransition I would NEVER see my […] children again,” “My father very much wanted me to desist,” and “Parents constantly encouraging me to detransition.” Five participants expressed societal pressure to detransition as expressed in the following quotes: “I did not pass, I was mocked in public, I could not get a job. It was not ok to be trans” and “Well, I mean basically the entire world was against me transitioning, so yeah.” One participant felt pressured by doctors and another one from a blog.

Detransition steps. Table 6 shows data about the social, medical, and surgical steps participants took to detransition. Nearly all participants medically detransitioned by ceasing cross-sex hormones (95.0%). Social detransition steps were also common and included returning to the use of previously used pronouns (63.0%) and birth names (33.0%) and changing one’s clothes and hair presentations (48.0%). Surgical detransition steps were less common (9.0%).

**Table 6:** Social, medical, and surgical detransition steps

	*N* (%)
Social detransition*	
Previous pronouns	63 (63.0%)
Clothes/hair/makeup	48 (48.0%)
Birth name	33 (33.0%)
New name (not birth name)	24 (24.0%)
None of the above	2 (2.0%)
Medical detransition*	
Stopped cross-sex hormones	95 (95.0%)
Stopped puberty blockers	4 (4.0%)
Started hormones consistent with natal sex	14 (14.0%)
Natal male	
Stopped anti-androgens	17 (54.8%)
Surgical detransition*	
Surgery to reverse changes from transition	9 (9.0%)

*May select more than one answer

Finding better ways of coping with gender dysphoria**.** Participants were asked to select responses that that they considered to have been better ways for them to cope with their gender dysphoria. Responses included community (44.0%), mindfulness/meditation (41.0%), exercise (39.0%), therapy (24.0%), trauma work (24.0%), medication to treat a mental health condition (18.0%), and yoga (14.0%).

### Transition and Detransition Narratives

Several transition and detransition narratives emerged from the data. A sizable minority of participants (41.0%) expressed more than one narrative in their responses.

The *discrimination and external pressures to detransition* narrative was described by 29.0% of participants. Examples include: “I had to detransition in order to get a job”; “I was afraid of being homeless and unable to support myself”; “I felt much happier with myself but I couldn't go anywhere without being afraid. I passed okay but not perfectly. I was stared down and sneered at in the women's clothes section, I wouldn't dare use a public toilet because I'd find either violent men or women who wished an encounter with a violent man on me.”

A *nonbinary* narrative was expressed by 16.0% of participants. Some described that they discovered their nonbinary gender identity during their transition, as in the following quotes: “I still was uncomfortable with my body and figured I should stop and make sure I really wanted to keep going. I didn't and I decided I must be nonbinary, not FTM”; “Transitioning didn’t do what I thought I wanted it to. I had transitioned to the wrong gender. I still felt wrong. Then, I realized I was not male, but genderqueer. I detransitioned to suit my true identity.” And others described a consistent nonbinary identification, as in the following quote, “I identified the same way that I did before. I had gotten what I wanted out of HRT and was ready to stop taking it.” (Cross-sex hormones are sometimes referred to as “hormone replacement therapy” and abbreviated as HRT).

Three participants (3.0%) expressed the *retransition* narrative in open-text answers indicating that they had retransitioned, including the following quotes: “I am now transitioning for a second time”; I retransitioned after 5 years of detransitioning”; and “Anyway, I retransitioned over 10 years after detransitioning.”

Most participants (58.0%) expressed the *gender dysphoria was caused by trauma or a mental health condition* narrative which included endorsing the response options indicating that their gender dysphoria was caused by something specific, such as a trauma or a mental health condition. More than half of the participants (51.2%) responded that they believe that the process of transitioning delayed or prevented them from dealing with or being treated for trauma or a mental health condition. The following are example quotes that were in response to why participants chose to detransition: “I slowly began addressing the mental health conditions and traumatic experiences that caused such a severe disconnect between myself and my body…”; “I was starting to become critical of transition because I felt that many people were doing it out of self-hatred and started to realize that applied to me as well”; “I was deeply uncomfortable with my secondary sex characteristics, which I now understand was a result of childhood trauma and associating my secondary sex characteristics with those events.”

Despite the absence of any questions about this topic in the survey, nearly a quarter (23.0%) of the participants expressed the *internalized homophobia and difficulty accepting oneself as lesbian, gay, or bisexual* narrative by spontaneously describing that these experiences were instrumental to their gender dysphoria, their desire to transition, and their detransition. All of the participants in this category indicated that they were either same-sex attracted exclusively or were same-sex attracted in combination with opposite-sex attraction (such as bisexual, pansexual, etc.). The following responses were written in as “other” for the question about why participants transitioned: “Transitioning to male would mean my attraction to girls would be ‘normal’”; “being a ‘gay trans man’ (female dating other females) felt better than being a lesbian, less shameful”; “I felt being the opposite gender would make my repressed same-sex attraction less scary”; “I didn't want to be a gay man.” Some participants described that it took time for them to gain an understanding of themselves as lesbian, gay, or bisexual as seen in the following: “At the time I was trying to figure out my identity and felt very male and thought I was transgender. I later discovered that I was a lesbian…”; and “Well, after deep discovery, I realized I was a gay man and realized that a sexual trauma after puberty might [have] confused my thought. I wanted to live as a gay man again.” Several natal female respondents expressed that seeing other butch lesbians would have been helpful to them as shown by the following: “What would have helped me is being able to access women's community, specifically lesbian community. I needed access to diverse female role-models and mentors, especially other butch women.”

The *social influence* narrative was identified where participants added information to the question about if they had felt pressured to transition and the response described pressure from a person or people. One-fifth (20.0%) of participants expressed that they felt pressured by a person or people to transition. Example quotes for social influence were described in a previous section.

Of the natal females, 7.2% expressed the *misogyny* narrative. Example quotes include: “…I realized how much of it [dysphoria] may have been caused by internalized misogyny and homophobia”; “Finally realizing there’s nothing wrong or disgusting or weak about being female”; and “My transition was a desperate attempt to distance myself from womanhood and femaleness due to internalized lesbophobia and misogyny combined with a history of sexual trauma.”

### After Detransition

**Fig. 1:**
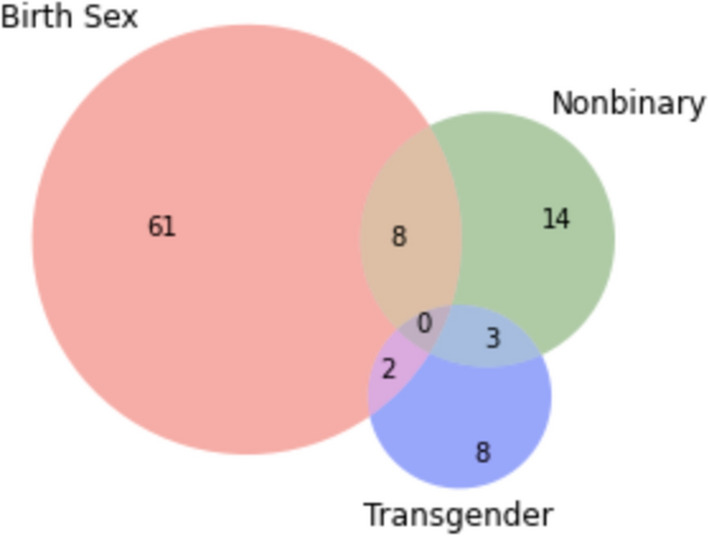
Distribution of participants’ current gender identification (after detransition) (n = 100). *Notes*: The sum of the numbers appearing in the “Birth Sex” circle indicates the number of participants who returned to identifying with their birth sex (71)—either as birth sex alone (61) or birth sex in addition to a second identification (10) represented in the overlap between two circles. For example, eight participants identify as their birth sex and as nonbinary. The sum of the numbers appearing in the “Nonbinary” circle indicates the number of participants who identify as nonbinary (25)—either as nonbinary alone (14) or nonbinary in addition to a second identification (11). The sum of the numbers appearing in the “Transgender” circle indicates the number of participants who identify as transgender (13)—either as transgender alone (8) or transgender in addition to a second identification (5). Four participants had responses that did not fit the categories above and were coded as “other”

Disposition. At the time of survey completion, most participants had returned to identifying solely as their birth sex (61.0%) with an additional 10.0% identifying as their birth sex plus another identification. Fourteen percent of the participants identified solely as nonbinary with an additional 11.0% identifying as nonbinary plus a second identification. Eight percent of the participants identified solely as transgender with an additional 5.0% identifying as transgender plus another identification. Four percent of the responses did not fit into the above categories and were coded as “other.” Figure 1 illustrates the distribution of participants’ current gender identification (post-detransition). Only 24.0% of participants had informed the doctor or clinic that facilitated their transitions that they had detransitioned.

Self-appraisal of past transgender identification. Table 7 presents the data for responses endorsed by participants to reflect how they feel currently about having identified as transgender in the past. The statements most frequently selected included: “I thought gender dysphoria was the best explanation for what I was feeling” (57.0%), “My gender dysphoria was similar to the gender dysphoria of those who remain transitioned” (42.0%), “What I thought were feelings of being transgender actually were the result of trauma” (36.0%), “What I thought were feelings of being transgender actually were the result of a mental health condition” (36.0%).

**Table 7:** Self-appraisal of past transgender identification

	Natal female *N* (%) *N* = 69	Natal male *N* (%) *N* = 31
Self-appraisal about identifying as transgender in the past*		
I thought gender dysphoria was the best explanation for what I was feeling	39 (56.5%)	18 (58.1%)
My gender dysphoria was similar to the gender dysphoria of those who remain transitioned	32 (46.4%)	10 (32.3%)
What I thought were feelings of being transgender actually were the result of trauma	31 (44.9%)	5 (16.1%)
What I thought were feelings of being transgender actually were the result of a mental health condition	28 (40.6%)	8 (25.8%)
Someone else told me that the feelings I was having meant that I was transgender and I believed them	25 (36.2%)	10 (32.3%)
I still identify as transgender	20 (29.0%)	10 (32.3%)
I believed I was transgender then, but I was mistaken	16 (23.2%)	6 (19.4%)
I was transgender then but I am not transgender now	15 (21.7%)	7 (22.6%)
I formerly identified as transgender and now identify as genderqueer/nonbinary	12 (17.4%)	5 (16.1)
My gender dysphoria was different from the gender dysphoria of those who remain transitioned	11 (15.9%)	4 (12.9%)
I was never transgender	8 (11.6%)	3 (9.7%)
I thought I had gender dysphoria but I was mistaken	4 (5.8%)	4 (12.9%)
I never had gender dysphoria	1 (1.4)	2 (6.5%)
N/A as I did not identify as transgender in the past	0 (0%)	1 (3.2%)
Other	18 (26.1%)	5 (16.1%)

*May select more than one answer

Self-appraisal of transition and detransition. When asked to select which statement best reflects their feelings about their transition, nearly a third (30.0%) indicated that they wish they had never transitioned while 11.0% indicated they were glad they transitioned. Some (34.0%) selected the statement that transition “was a necessary part of [their] journey” but others (21.0%) indicated that the process of transitioning distracted them from what they should have been doing. Responses about whether transition helped or harmed them were also complicated. While 50.5% selected answers consistent with being both helped and harmed, 32.3% indicated that they were only harmed and 17.2% indicated that they were only helped. The majority of respondents were dissatisfied with their decision to transition (69.7%) and satisfied with their decision to detransition (84.7%). At least some amount of transition regret was common (79.8%) and nearly half (49.5%) reported strong or very strong regret. Most respondents (64.6%) indicated that if they knew then what they know now, they would not have chosen to transition.

## Discussion

This study was designed to explore the experiences of individuals who obtained medical and surgical treatment for gender dysphoria and then detransitioned by discontinuing the medications or having surgery to reverse the changes from transition. The findings of this study, however, should not be assumed to be representative of all individuals who detransition. Although this study further documents that detransitioners exist, the prevalence of detransition as an outcome of transition is unknown. Only a small percentage of detransitioners (24.0%) informed the clinicians and clinics that facilitated their transitions that they had detransitioned. Therefore, clinic rates of detransition are likely to be underestimated and gender transition specialists may be unaware of how many of their own patients have detransitioned, particularly for patients who are no longer under their care.

This research demonstrates that the experiences of individuals who detransition are varied and the reasons for detransition are complex. Nearly all participants identified as transgender or nonbinary at the start of their transition and most sought transition because they did not want to be associated with their natal sex, their bodies felt wrong the way they were, and they believed that transition was the only option to relieve their distress. Some were helped by transition and only detransitioned because they were pressured to do so by people in their lives, society, or because they had medical complications. Some were harmed by transition and detransitioned because they concluded that their gender dysphoria was caused by trauma, a mental health condition, internalized homophobia, or misogyny—conditions that are not likely to be resolved with transition. These findings highlight the complexity of gender dysphoria and suggest that, in some cases, failure to explore co-morbidities and the context in which the gender dysphoria emerged can lead to misdiagnosis, missed diagnoses, and inappropriate gender transition. Some individuals detransitioned because their gender dysphoria resolved, because they found better ways to address their symptoms, or because their personal definitions of male and female changed and they became comfortable identifying as their natal sex.

The study sample was predominantly young natal females, many of whom experienced late-onset gender dysphoria which mirrors the recent, striking changes in the demographics of gender dysphoric youth seeking care as well as the youth described by their parents in Littman (2018) (see also Aitken et al., 2015; de Graaf et al., 2018; Zucker, 2019). Concerns have been raised that this new cohort of gender dysphoric individuals is unlike previous cohorts. Professionals have started to call for caution before treating this cohort with interventions with permanent effects because the etiologies, desistance and persistence rates, expected duration of symptoms, and whether this new population is helped or harmed by gender transition is still unknown (D’Angelo et al., 2021; Kaltiala-Heino et al., 2018). The natal females and natal males in this sample differed on several dimensions, including that natal females were younger than natal males when they sought transition, when they decided to detransition, and at the time of survey completion. Natal females were more likely than natal males to have experienced a trauma less than one year before the onset of their gender dysphoria and were more likely to have felt pressured to transition. Compared to natal males, natal females remained transitioned for a shorter duration of time before deciding to detransition. Additionally, natal females transitioned more recently than natal males, so their experiences may vary due to changing trends in the clinical management of gender dysphoria and the cultural settings in which they became gender dysphoric.

The study findings covered a wide range of detransition experiences that are consistent with the diversity of experiences described in previously published clinical case reports and case series. Overlap of findings include: transition regret; absence of transition regret; re-identification with birth sex; continued identification as transgender; improvement or worsening of well-being with transition; retransitioning; detransitioning due to external social pressures; nonbinary identification; and recognizing and accepting oneself as homosexual or bisexual (D’Angelo, 2018; Djordjevic et al., 2016; Levine, 2018; Pazos Guerra et al., 2020; Turban & Keuroghlian, 2018; Turban et al., 2021; Vandenbussche, 2021). The population in this study is similar to the population in Vandenbussche in that both were predominantly natal females in their mid-20s. Because the current study recruited in 2016–2017 and Vandenbussche recruited in 2019, the similar mean age of participants may reflect the age of individuals who can be reached in online detransitioner communities. Several findings in this study were consistent with Vandenbussche’s findings, including similar reasons for detransition (realizing that their gender dysphoria was related to other issues, finding alternatives to address gender dysphoria, gender dysphoria resolved, etc.). Although these two studies were recruited in different years, had different eligibility criteria, and included participants from several countries, it is possible that there may be some overlap of study populations.

The current study findings provide additional insight into the complex relationships between internalized homophobia, gender dysphoria, and desire to transition. Contrary to arguments against the potential role of homophobia in gender transitions (Ashley, 2020), participants reported that their own gender dysphoria and desire to transition stemmed from the discomfort they felt about being same-sex attracted, their desire to not be gay, and the difficulties that they had accepting themselves as lesbian, gay or bisexual. For these individuals, exploring their distress and discomfort around sexual orientation issues may have been more helpful to them than medical and surgical transition or at least an important part of exploration before making the decision to transition. This research adds to the existing evidence that gender dysphoria can be temporary (Ristori & Steensma, 2016; Singh et al., 2021; Zucker, 2018). It has been established that the most likely outcome for prepubertal youth with gender dysphoria is to develop into lesbian, gay, bisexual (LGB) (non-transgender) adults (Ristori & Steensma, 2016; Singh et al., 2021; Wallien & Cohen-Kettenis, 2008; Zucker, 2018). And, temporary gender dysphoria may be a common part of LGB identity development (Korte et al., 2008; Patterson, 2018). Therefore, intervening too soon to medicalize gender dysphoric youth risks iatrogenically derailing the development of youth who would otherwise grow up to be LGB non-transgender adults. Participants who detransitioned because they became comfortable identifying as their natal sex and because their gender dysphoria resolved further support that gender dysphoria is not always permanent.

The data in this study strengthen, with first-hand accounts, the rapid-onset gender dysphoria (ROGD) hypotheses which, briefly stated, are that psychosocial factors (such as trauma, mental health conditions, maladaptive coping mechanisms, internalized homophobia, and social influence) can cause or contribute to the development of gender dysphoria in some individuals (Littman, 2018). Littman also postulated that certain beliefs could be spread by peer contagion, including the belief that a wide range of symptoms should be interpreted as gender dysphoria (and proof of being transgender) and the belief that transition is the only solution to relieve distress. The current study supports the potential role of psychosocial factors in the development of gender dysphoria and further suggests, by participant responses that transitioning prevented or delayed them from addressing their underlying conditions, that maladaptive coping mechanisms may be relevant for some individuals. The potential role of social influence is demonstrated as well. First, when respondents were asked to describe how they currently feel about having identified as transgender in the past, more than a third endorsed the option, “Someone told me that the feelings I was having meant that I was transgender, and I believed them.” Second, a subset of participants experienced the unique friendship group dynamics reported in Littman where peer groups mocked people who were not transgender and popularity within the friend group increased when respondents announced their plan to transition. Additionally, respondents identified several social sources that encouraged them to believe that transitioning would help them including: YouTube transition videos, blogs, Tumblr, and online communities. And finally, 20.0% of participants felt pressured to transition by social sources that included friends, partners, and society. More research is needed to further explore these hypotheses.

The current study and the Turban et al. (2021) analysis of the USTS data share some similarities and differences. Similarities include the use of convenience samples, targeted recruitment, and anonymous data collection. The findings of Turban et al. (including external pressures to detransition and transgender identification after detransition) are a subset of the array of experiences described in the current study. The current study differed from James et al. (2016) and Turban et al. in that it enrolled participants based on the criterion of detransition after medical or surgical transition regardless of how they currently identified, recruited from communities with diverse perspectives about transition and detransition, used a precise definition for detransition that specifies the use of medication or surgery, and included answer options that were relevant to many different types of detransition experiences. In contrast, the USTS only enrolled transgender-identifying individuals regardless of whether they medically or surgically transitioned, recruited from communities likely to have similar perspectives about transition and detransition, and provided multiple choice answer options that were relevant to a narrower range of detransition experiences (James et al., 2016). Further, the definition used by the USTS for “detransitioned” (having “gone back to living as [their] sex assigned as birth, at least for a while”) is quite vague. Although Turban et al. provide valuable information about the subset of transgender-identifying people who may have detransitioned, the current study provides a more comprehensive view of individuals who detransition after medical or surgical transition.

Over the past 15 years, there have been substantial changes in the clinical approach to gender dysphoric patients notable for a shift from approaches that employ thorough evaluations and judicious use of medical and surgical transition (the watchful waiting or Dutch approach, the developmentally informed approach, and the medical model of care) to approaches with minimized or eliminated evaluation and liberal use of transition interventions (the affirmative approach and the informed consent model of care) (Cavanaugh et al., 2016; de Vries & Cohen-Kettenis, 2012; Meyer et al., 2002; Rafferty et al., 2018; Schulz, 2018; Zucker et al., 2012b). This trend is prominent in the U.S. where the American Academy of Pediatrics endorsed the affirmative approach in 2018 and Planned Parenthood currently uses the informed consent model to provide medical transition in more than 200 clinics in 35 states (Planned Parenthood, 2021; Rafferty et al., 2018). It is plausible that an unintended consequence of these clinical shifts may be an increase in people who detransition. Many participants in this study believe that they did not receive an adequate evaluation by a clinician before transition. The definition of “adequate evaluation” was not provided in the survey and may be open to respondent interpretation. But given the complexities of the gender dysphoria described in the current study, one might consider a low bar of “adequate” to be the exploration of factors that could be misinterpreted as non-temporary gender dysphoria as well as factors that could be underlying causes for gender dysphoria. The most recently emerging approach to gender dysphoria is called the “exploratory approach” which is a neutral psychotherapeutic approach to help individuals gain a deeper understanding of their gender distress and the factors contributing to their dysphoria (Churcher Clarke & Spiliadis, 2019; Spiliadis, 2019). The study’s findings suggest that an exploratory type of approach may have been beneficial to some of the respondents. Future research is needed to determine which patients are best treated by which approaches long term.

Patients considering medical and surgical interventions deserve accurate information about the risks, benefits, and alternatives to that treatment. In this sample, nearly half of the participants reported that the counseling they received about transition was overly positive about the benefits of transition and more than a quarter reported that the counseling was not negative enough about the risks. Several participants felt pressured to transition by their doctors and therapists. If these types of clinical interactions are verified, exploration is needed to determine the extent to which this situation occurs and what measures might be taken to ensure that clinicians provide patients with their options accurately and dispassionately.

There are several obstacles to obtaining accurate rates of detransition and desistance, including stigma and the low numbers of detransitioners who inform their clinicians that they detransitioned. One approach to bypass some of these barriers would be to incorporate non-judgmental questions about detransition and desistance into nationally representative surveys that collect health data. For example, the Behavioral Risk Factor Surveillance System contains an optional module about sexual orientation and gender identity that includes two questions to explore gender issues (Downing & Przedworski, 2018). By changing one existing question, “Do you consider yourself to be transgender?” into two questions, “Have you ever, at any point in your life, considered yourself to be transgender?” and “Do you currently consider yourself to be transgender?” and by adding a follow-up question if answers indicate past but not current transgender identification, “Did you ever take puberty blockers, cross-sex hormones, anti-androgens, or have any surgery as part of your transition?”, valuable information about desistance, detransition, and current transgender identification could be obtained. These types of questions may also be of use in clinical practice and electronic medical records. The information gained about rates of detransition and desistance would enhance transgender healthcare by aiding informed consent processes at the start of any medical or surgical transition.

One of the strengths of this study is that it is one of the largest samples of detransitioners to date. Other strengths include the use of a precise definition for detransition, enrollment of detransitioners regardless of their post-detransition gender identification, recruitment from communities with likely divergent views about transition and detransition, and collaboration with two individuals who had detransitioned which helped to create a survey instrument with questions relevant to a variety of detransition experiences and enhanced the recruitment efforts.

There are several limitations to this study that should be considered when interpreting the findings. Like Vandenbussche (2021), James et al. (2016), and Turban et al. (2021), this study used a cross-sectional design, anonymous surveying, and a convenience sample and therefore shares the same limitations that are inherent to these methodologies. These limitations include that conclusions about causation cannot be determined, identities of participants cannot be verified, and the findings of this study may not be generalizable to the entire population of people who detransition or to people outside of the countries where participants were from. Although this study reached out to communities with differing perspectives about transition and detransition, targeted recruitment and convenience samples always introduce the limitations associated with selection biases which should be addressed in future research. Finally, many of the participants in this study had less than ideal outcomes to their medical and surgical transitions, and it is possible that these experiences may have colored some of the responses.

Additional research is needed to determine the prevalence of detransition as an outcome of transition and to identify and meet the psychological and medical needs of the emerging detransitioned population. Because many individuals who detransition re-identify with their birth sex, are no longer connected to LGBT communities, and don’t return to gender clinics, future research about detransition needs to expand recruitment efforts beyond gender clinics and transgender communities. The development and testing of non-medical interventions for gender dysphoria could provide valuable options to be used as alternatives or in conjunction with medical and surgical treatments. Because of the potential for some to experience trauma, mental health conditions, internalized homophobia, and misogyny as gender dysphoria, research needs to be conducted on the evaluation process before transition to find approaches that respectfully and collaboratively explore factors that might contribute to gender-related distress. There continues to be an absence of long-term outcomes evidence for youth treated with medical and surgical transition and a lack of information about the trajectories of youth experiencing late-onset gender dysphoria–research is needed to address these gaps. Continued work is needed to reduce rigid gender roles, increase representation of gender stereotype nonconformity, and to address discrimination and social pressures exerted against people who are transgender, lesbian, gay, bisexual, and gender stereotype non-conforming.

### Conclusion

This study described individuals who, after transitioning with medications or surgery, have detransitioned. The prevalence of detransitioning after transition is unknown but is likely underestimated because most of the participants did not inform the doctors who facilitated their transitions that they had detransitioned. There is no single narrative to explain the experiences of all individuals who detransition and we should take care to avoid painting this population with a broad brush. Some detransitioners return to identifying with their birth sex, some assume (or maintain) a nonbinary identification, and some continue to identify as transgender. Some detransitioners regret transitioning and some do not. Some of the detransitioners reported experiences that support the ROGD hypotheses, including that their gender dysphoria began during or after puberty and that mental health issues, trauma, peers, social media, online communities, and difficulty accepting themselves as lesbian, gay, or bisexual were related to their gender dysphoria and desire to transition. Natal female and natal male detransitioners appear to have differences in their baseline characteristics and experiences and these differences should be further delineated. Future research about gender dysphoria and the outcomes of transition should consider the diversity of experiences and trajectories. More research is needed to determine how best to provide support and treatment for the long-term medical and psychological well-being of individuals who detransition. Findings about detransition should be used to improve our understanding of gender dysphoria and to better inform the processes of evaluation, counseling, and informed consent for individuals who are contemplating transition.

## Supplementary Information

Below is the link to the electronic supplementary material.

Supplementary file1 (DOCX 38 kb)

## Footnotes

1 A search of the word “detransition” in YouTube can be filtered by date of upload. https://www.youtube.com/results?search_query=%22detransition%22&sp=CAI%253D22.

2 The debate about the terminologies used to describe an individual’s sex (including “assigned sex at birth,” “biological sex,” “natal sex,” “birth sex,” “sex,” etc.) is far from settled. Although some professionals have argued for the use of “assigned sex at birth,” others argue that this terminology is misleading and not consistent with the events that occur at birth and prior to birth (Bouman et al., 2017; Byng et al., 2018; Dahlen, 2020; Griffin et al., 2020). Supporting the unsettled nature of the discussion, I received conflicting comments from the reviewers of this manuscript about my selection of natal sex terms–one reviewer asked that I justify my preference for natal sex over the other terminologies; another reviewer expressed support for my use of natal sex. I prefer to use “natal sex” and “birth sex” because they are accurate and objective. Further, I propose that “natal sex” and “birth sex” might be seen as reasonable, polite compromise terms between “biological sex” and “assigned sex at birth.”

## References

[CR2] Aitken M, Steensma TD, Blanchard R, VanderLaan DP, Wood H, Fuentes A, Spegg C, Wasserman L, Ames M, Fitzsimmons CL, Leef JH, Lishak V, Reim E, Takagi A, Vinik J, Wreford J, Cohen-Kettenis PT, de Vries ALC, Kreukels BPC, Zucker KJ (2015). Evidence for an altered sex ratio in clinic-referred adolescents with gender dysphoria. Journal of Sexual Medicine.

[CR3] Anonymous. (2017). Experience: I regret transitioning. *The Guardian*. https://www.theguardian.com/lifeandstyle/2017/feb/03/experience-i-regret-transitioning

[CR4] Ashley F (2020). Homophobia, conversion therapy, and care models for trans youth: Defending the gender-affirmative approach. Journal of LGBT Youth.

[CR5] Blanchard R (1985). Typology of male-to-female transsexualism. Archives of Sexual Behavior.

[CR6] Blanchard R (1989). The classification and labeling of nonhomosexual gender dysphorias. Archives of Sexual Behavior.

[CR7] Blanchard R, Clemmensen LH, Steiner BW (1987). Heterosexual and homosexual gender dysphoria. Archives of Sexual Behavior.

[CR8] Bouman WP, Schwend AS, Motmans J, Smiley A, Safer JD, Deutsch MB, Adams NJ, Winter S (2017). Language and trans health [Editorial]. International Journal of Transgenderism.

[CR9] Bridge, L. (2020). Detransitioners are living proof the practices surrounding “trans kids” need to be questioned. *Feminist Current.* https://www.feministcurrent.com/2020/01/09/detransitioners-are-living-proof-the-practices-surrounding-trans-kids-need-be-questioned/

[CR10] Butler C, Hutchinson A (2020). Debate: The pressing need for research and services for gender desisters/detransitioners. Child and Adolescent Mental Health.

[CR11] Byng, R., Bewley, S., Clifford, D., & McCartney, M. (2018). Redesigning gender identity services: An opportunity to generate evidence. *British Medical Journal*, *363*. 10.1136/bmj.k4490 10.1136/bmj.k4490 30373856

[CR12] Callahan C, Brunskell-Evans H, Moore M (2018). Unheard voices of detransitioners. Transgender children and young people: Born in your own body.

[CR13] Cavanaugh T, Hopwood R, Lambert C (2016). Informed consent in the medical care of transgender and gender-nonconforming patients. AMA Journal of Ethics.

[CR14] Churcher Clarke A, Spiliadis A (2019). ‘Taking the lid off the box’: The value of extended clinical assessment for adolescents presenting with gender identity difficulties. Clinical Child Psychology and Psychiatry.

[CR15] Dahlen S (2020). De-sexing the medical record? An examination of sex versus gender identity in the General Medical Council’s trans healthcare ethical advice. The New Bioethics.

[CR16] D’Angelo R (2018). Psychiatry’s ethical involvement in gender-affirming care. Australasian Psychiatry.

[CR17] D’Angelo R, Syrulnik E, Ayad S, Marchiano L, Kenny DT, Clarke P (2021). One size does not fit all: In support of psychotherapy for gender dysphoria [Letter to the Editor]. Archives of Sexual Behavior.

[CR18] de Graaf NM, Giovanardi G, Zitz C, Carmichael P (2018). Sex ratio in children and adolescents referred to the Gender Identity Development Service in the UK (2009–2016) [Letter to the Editor]. Archives of Sexual Behavior.

[CR19] de Vries ALC, Cohen-Kettenis PT (2012). Clinical management of gender dysphoria in children and adolescents: The Dutch approach. Journal of Homosexuality.

[CR20] Djordjevic ML, Bizic MR, Duisin D, Bouman M-B, Buncamper M (2016). Reversal surgery in regretful male-to-female transsexuals after sex reassignment surgery. Journal of Sexual Medicine.

[CR21] Downing JM, Przedworski JM (2018). Health of transgender adults in the U.S., 2014–2016. American Journal of Preventive Medicine.

[CR22] Entwistle K (2021). Debate: Reality check–Detransitioner’s testimonies require us to rethink gender dysphoria. Child and Adolescent Mental Health.

[CR1] 4thwavenow. (2016). *In praise of gatekeepers: An interview with a former teen client of TransActive Gender Center*. https://4thwavenow.com/2016/04/21/in-praise-of-gatekeepers-an-interview-with-a-former-teen-client-of-transactive-gender-center/

[CR23] Griffin L, Clyde K, Byng R, Bewley S (2020). Sex, gender and gender identity: A re-evaluation of the evidence. BJPsych Bulletin.

[CR24] Herzog, K. (2017). The detransitioners: They were transgender until they weren’t. *The Stranger*. https://www.thestranger.com/features/2017/06/28/25252342/the-detransitioners-they-were-transgender-until-they-werent

[CR25] James SE, Herman JL, Rankin S, Keisling M, Mottet L, Anafi M (2016). The Report of the 2015 U.S. Transgender Survey.

[CR26] Kaltiala-Heino R, Bergman H, Työläjärvi M, Frisen L (2018). Gender dysphoria in adolescence: Current perspectives. Adolescent Health, Medicine and Therapeutics.

[CR27] Korte A, Goecker D, Krude H, Lehmkuhl U, Grüters-Kieslich A, Beier KM (2008). Gender identity disorders in childhood and adolescence currently debated concepts and treatment strategies. Deutsches Aerzteblatt Online.

[CR28] Kuiper AJ, Cohen-Kettenis PT (1998). Gender role reversal among postoperative transsexuals. International Journal of Transgenderism.

[CR29] Levine SB (2018). Transitioning back to maleness. Archives of Sexual Behavior.

[CR30] Littman L (2018). Parent reports of adolescents and young adults perceived to show signs of a rapid onset of gender dysphoria. PLoS ONE.

[CR31] Marchiano L (2017). Outbreak: On transgender teens and psychic epidemics. Psychological Perspectives: A Quarterly Journal of Jungian Thought.

[CR32] Marchiano, L. (2020). The ranks of gender detransitioners are growing. We need to understand why. *Quillette*. https://quillette.com/2020/01/02/the-ranks-of-gender-detransitioners-are-growing-we-need-to-understand-why/

[CR33] McCann, C. (2017). When girls won’t be girls. *The Economist*. https://www.economist.com/1843/2017/09/28/when-girls-wont-be-girls

[CR34] Meyer W, Bockting WO, Cohen-Kettenis P, Coleman E, Diceglie D, Devor H, Gooren L, Hage JJ, Kirk S, Kuiper B, Laub D, Lawrence A, Menard Y, Patton J, Schaefer L, Webb A, Wheeler CC (2002). The Harry Benjamin International Gender Dysphoria Association’s standards of care for gender identity disorders, Sixth Version. Journal of Psychology & Human Sexuality.

[CR35] Patterson T (2018). Unconscious homophobia and the rise of the transgender movement. Psychodynamic Practice.

[CR36] Pazos Guerra M, Gómez Balaguer M, Gomes Porras M, Hurtado Murillo F, Solá Izquierdo E, Morillas Ariño C (2020). Transexualidad: Transiciones, detransiciones y arrepentimientos en España. Endocrinología, Diabetes y Nutrición.

[CR37] Pique Resilience Project. (2019). https://www.piqueresproject.com/

[CR38] Planned Parenthood. (2021). *What do I need to know about trans health care?* https://www.plannedparenthood.org/learn/gender-identity/transgender/what-do-i-need-know-about-trans-health-care

[CR39] Rafferty, J., Committee on Psychosocial Aspects of Child and Family Health, Committee on Adolescence, & Section on Lesbian, Gay, Bisexual, and Transgender Health and Wellness. (2018). Ensuring comprehensive care and support for transgender and gender-diverse children and adolescents. *Pediatrics*, *142*(4), e20182162. 10.1542/peds.2018-2162 10.1542/peds.2018-2162 30224363

[CR40] r/detrans. (2019). *R/detrans subreddit survey update!* [Reddit]. https://www.reddit.com/r/detrans/comments/azj8xd/subreddit_survey_update/

[CR41] r/detrans. (2020). [Reddit]. https://www.reddit.com/r/detrans/

[CR42] Ristori J, Steensma TD (2016). Gender dysphoria in childhood. International Review of Psychiatry.

[CR43] Schulz SL (2018). The informed consent model of transgender care: An alternative to the diagnosis of gender dysphoria. Journal of Humanistic Psychology.

[CR44] Singh D, Bradley SJ, Zucker KJ (2021). A follow-up study of boys with gender identity disorder. Frontiers in Psychiatry.

[CR45] Spiliadis A (2019). Towards a gender exploratory model: Slowing things down, opening things up and exploring identity development. Metalogos Systemic Therapy Journal.

[CR46] Steensma TD, Kreukels BPC, de Vries ALC, Cohen-Kettenis PT (2013). Gender identity development in adolescence. Hormones and Behavior.

[CR47] Stella, C. (2016). *Female detransition and reidentification: Survey results and interpretation* [Tumblr]. http://guideonragingstars.tumblr.com/post/149877706175/female-detransition-and-reidentification-survey

[CR48] The Detransition Advocacy Network. (2020). https://www.detransadv.com

[CR49] Tracey, M. (2020). *Why all this trans stuff?* YouTube. https://youtu.be/r57wGbiK3U8

[CR50] Turban JL, Keuroghlian AS (2018). Dynamic gender presentations: Understanding transition and “de-transition” among transgender youth. Journal of the American Academy of Child and Adolescent Psychiatry.

[CR51] Turban JL, Loo SS, Almazan AN, Keuroghlian AS (2021). Factors leading to “detransition” among transgender and gender diverse people in the United States: A mixed-methods analysis. LGBT Health.

[CR52] twitter.com/ftmdetransed, & twitter.com/radfemjourney. (2019). Our voices our selves—Amplifying the voices of detransitioned women. In M. Moore & H. Brunskell-Evans (Eds.), *Inventing transgender children and young people* (pp. 167–174). Cambridge Scholars Publishing.

[CR53] upperhandMARS. (2020). *Desist to exist as Chiara*. YouTube. https://www.youtube.com/watch?v=rLfTrTRnIRk

[CR54] Vandenbussche E (2021). Detransition-related needs and support: A cross-sectional online survey. Journal of Homosexuality.

[CR55] Wallien MSC, Cohen-Kettenis PT (2008). Psychosexual outcome of gender-dysphoric children. Journal of the American Academy of Child and Adolescent Psychiatry.

[CR56] Zucker KJ (2018). The myth of persistence: Response to “A critical commentary on follow-up studies and ‘desistance’ theories about transgender and gender non-conforming children” by Temple Newhook et al. (2018). International Journal of Transgenderism.

[CR57] Zucker KJ (2019). Adolescents with gender dysphoria: Reflections on some contemporary clinical and research issues. Archives of Sexual Behavior.

[CR58] Zucker KJ, Bradley SJ (1995). Gender identity disorder and psychosexual problems in children and adolescents.

[CR59] Zucker KJ, Bradley SJ, Owen-Anderson A, Kibblewhite SJ, Wood H, Singh D, Choi K (2012). Demographics, behavior problems, and psychosexual characteristics of adolescents with gender identity disorder or transvestic fetishism. Journal of Sex & Marital Therapy.

[CR60] Zucker KJ, Wood H, Singh D, Bradley SJ (2012). A developmental, biopsychosocial model for the treatment of children with gender identity disorder. Journal of Homosexuality.

