# Supplementary material for: Individuals Treated for Gender Dysphoria with Medical and/or Surgical Transition Who Subsequently Detransitioned: A Survey of 100 Detransitioners
Source: Arch Sex Behav. 2021 Oct 19;50(8):3353–69. doi: 10.1007/s10508-021-02163-w (PMC8604821; doi:10.1007/s10508-021-02163-w)
Supplement: Supplementary file 1 — Supplementary file1 (DOCX 38 kb) [file 10508_2021_2163_MOESM1_ESM.docx]

**Supplemental Document**

*Survey questions used for narratives*

42. In your own words, please describe why you decided to transition, how you identified at that time, and what you hoped would happen with your transition.

43. Which of the following were reasons that you wanted to transition? (please check all that apply)

- I thought transitioning would eliminate my gender dysphoria
- I thought transitioning would lesson my gender dysphoria
- I thought that transitioning was my only option to feel better
- I thought it would reduce gender related harassment or trauma I was experiencing.
- I didn't want to be associated with my natal sex/natal gender.
- I saw myself as a member of the target gender.
- It made me uncomfortable to be perceived romantically/sexually as a member of my natal sex/natal gender.
- I identified with the target gender.
- I felt I would fit in better with the target gender.
- I felt I would be treated better if perceived as the target gender.
- I felt I would be more socially acceptable as a member of the target gender.
- I had erotic reasons for wanting to transition
- My body felt wrong to me the way it was.
- I wanted others to perceive me as the target gender.
- I felt I would become my true self
- Other (please specify)

46. Do any of the following statements describe how you feel now about identifying as transgender in the past? (choose all that apply)

- I believed that I was transgender then, but was mistaken.
- I was transgender then but I am not transgender now.
- I was never transgender
- I still identify as transgender
- I formerly identified as transgender and now identify as genderqueer/non-binary
- Someone else told me that the feelings I was having meant that I was transgender and I believed them.
- What I thought were feelings of being transgender actually were the result of trauma.
- What I thought were feelings of being transgender actually were the result of a mental health condition.
- I thought gender dysphoria was the best explanation for what I was feeling
- I thought I had gender dysphoria but I was mistaken
- I never had gender dysphoria
- My gender dysphoria was different than the gender dysphoria of those who remain transitioned
- My gender dysphoria was similar than the gender dysphoria of those who remain transitioned.
- N/A as I did not identify as transgender in the past
- Other (please specify)

73. What kind of evaluation, counseling and information do you wish you had received before transition?

74. Is there anything in your personal history that you think should have prevented you from receiving medication or surgery for transition?

- Yes
- No
  If you would like to explain you may do so here

85. At any point, did you feel pressured to transition?

- Yes
- No
  If yes, please describe.

90. Do you believe that the process of transitioning prevented or delayed you from dealing with or being treated for a trauma or mental health condition?

- N/A
- Yes
- No
- Don't know

If you'd like to add more information, you may do so here.

95. In your own words, please describe why you chose to detransition, how you identified when you chose to de-transition, and what you hoped for in de-transitioning.

96. Do any of the following reasons apply for your reason(s) for detransitioning? (choose all that apply)

- My mental health was worse while transitioning
- My mental health did not improve while transitioning
- I discovered that my gender dysphoria was caused by something specific (ex, trauma, abuse, mental health condition)
- I resolved the specific issue that was the cause of my gender dysphoria.
- My physical health was worse while transitioning
- My physical health did not improve while transitioning.
- I had medical complications from transitioning.
- I was concerned about potential medical complications from transitioning.
- My gender dysphoria resolved
- I was dissatisfied by the physical results of the transition/felt that the change was too much
- I was dissatisfied with the physical results of transition/felt the change was not enough
- My personal definition of female or male changed and I became more comfortable identifying as my natal sex.
- financial concerns around paying for transition care
- I realized that my desire to transition was erotically motivated
- I felt discriminated against
- I found more effective ways to help my gender dysphoria
- Other (please specify)

98. At any time, did you feel pressured to de-transition?

- Yes
- No
  If yes, please describe.

102. If you found better ways to cope with gender dysphoria, please describe what was helpful to you.

106. How do you identify now?

- Female
- Male
- Nonbinary/Genderqueer
- Trans Man/FTM
- Trans Woman/MTF
- None of the above Other (please specify)

115. If you would like to, please tell us more about any aspect of your experience or anything you think might be helpful for our research.

**Table S1**

*Sources that encouraged participants to believe that transition would help them*

|  |  | Natal Female  N(%)  N=69 | Natal Male  N(%)  N=31 |
| --- | --- | --- | --- |
| Sources that encouraged the participants to believe that transitioning would help them * |  |  |  |
|  | YouTube transition videos | 41 (59.4%) | 7 (22.6%) |
|  | Blogs | 33 (47.8%) | 13 (41.9%) |
|  | Tumblr | 40 (58.0%) | 5 (16.1%) |
|  | A community of people that they met online | 35 (50.7%) | 8 (25.8%) |
|  | Therapist | 27 (39.1%) | 10 (32.3%) |
|  | A person they met online | 18 (26.1%) | 12 (38.7%) |
|  | A person they know in person (not online) | 22 (31.9%) | 6 (19.4%) |
|  | A group of friends known in person (not online) | 22 (31.9%) | 5 (16.1%) |
|  | Reddit | 14 (20.3%) | 7 (22.6%) |
|  | Facebook | 16 (23.2%) | 1 (3.2%) |
|  | Gay Straight Alliance (GSA) | 12 (17.4%) | 2 (6.5%) |
|  | Group therapy setting | 4 (5.8%) | 3 (9.7%) |
|  | DeviantART | 5 (7.2%) | 1 (3.2%) |
|  | Cosplay communities | 5 (7.2%) | 0 (0%) |
|  | Religious community | 2 (2.9%) | 0 (0%) |
|  | Gaming community | 2 (2.9%) | 0 (0%) |
|  | Homeschooling community | 1 (1.4%) | 0 (0%) |
|  | Family member | 1 (1.4%) | 0 (0%) |
|  | None of the above | 3 (4.3%) | 9 (29.0%) |
|  | Other | 11 (15.9%) | 4 (12.9%) |

* may select more than one answer

**Table S2**

*Types of clinicians and practices seen for gender dysphoria and transition*

|  |  | Natal Female  N (%)  N=69 | Natal Male  N (%)  N=31 |
| --- | --- | --- | --- |
| First type of doctor or mental health professional that participant visited for gender dysphoria or wanting to transition* |  |  |  |
|  | Psychiatrist or psychologist | 34 (49.3%) | 19 (61.3%) |
|  | Primary care physician (general practice, family medicine, internal medicine, pediatrician, obstetrician-gynecologist) | 24 (34.8%) | 10 (32.3%) |
|  | Counselor (LCSW, LPC, MFT) | 23 (33.3%) | 9 (20.0%) |
|  | Endocrinologist | 10 (14.5%) | 7 (22.6%) |
|  | Nurse practitioner | 2 (2.9%) | 2 (6.5%) |
|  | Surgeon | 2 (2.9%) | 1 (3.2%) |
|  | Not sure | 6 (8.7%) | 0 (0%) |
| Type of practice visited for transition * |  |  |  |
|  | Gender clinic (any) | 30 (43.5%) | 15 (48.4%) |
|  | Gender clinic (informed consent, specifically) | 17 (24.6%) | 3 (9.7%) |
|  | Private doctor’s office | 17 (24.6%) | 11 (35.5%) |
|  | Group practice (single or multispecialty) | 20 (29.0%) | 6 (19.4%) |
|  | Mental health clinic | 9 (13.0%) | 4 (12.9%) |
|  | Other | 7 (10.1%) | 1 (3.2%) |

* may select more than one answer

**Table S3**

*Sources that encouraged participants to believe that detransition would help them*

| Sources that encouraged participants to believe that detransitioning would help them* |  | Natal Female  N (%)  N=69 | Natal Male  N (%)  N=31 |
| --- | --- | --- | --- |
|  | Blogs | 28 (40.6%) | 9 (29.0%) |
|  | Tumblr | 30 (43.5%) | 5 (16.1%) |
|  | YouTube detransition videos | 15 (21.7%) | 8 (25.8%) |
|  | A person they know in person (not online) | 15 (21.7%) | 6 (19.4%) |
|  | A community of people that they met online | 15 (21.7%) | 4 (12.9%) |
|  | A person they met online | 9 (13.0%) | 4 (12.9%) |
|  | Reddit | 5 (7.2%) | 6 (19.4%) |
|  | A group of friends known in person (not online) | 8 (11.6%) | 2 (6.5%) |
|  | Facebook | 5 (7.2%) | 2 (6.5%) |
|  | Family member | 4 (5.8%) | 2 (6.5%) |
|  | Therapist | 2 (2.9%) | 4 (12.9%) |
|  | Religious community | 2 (2.9%) | 0 (0%) |
|  | Group therapy setting | 1 (1.4%) | 0 (0%) |
|  | Gaming community | 1 (1.4%) | 0 (0%) |
|  | None of the above | 19 (27.5%) | 10 (32.3%) |
|  | Other | 10 (14.5%) | 7 (22.6%) |

* may select more than one answer
